# Loss of exosomal LncRNA HCG15 prevents acute myocardial ischemic injury through the NF-κB/p65 and p38 pathways

**DOI:** 10.1038/s41419-021-04281-8

**Published:** 2021-10-27

**Authors:** Beiyou Lin, Xi Chen, Chuanghong Lu, Jianjun Xu, Yumin Qiu, Xin Liu, Haoyu Song, Ang Chen, Jie Xiong, Kun Wang, Yuan Yuan, Lile Shi, Lintao Zhong, Xiaofei Jiang

**Affiliations:** 1 Department of Cardiology, Zhuhai People’s Hospital (Zhuhai Hospital Affiliated with Jinan University), Zhuhai, China; 2 Department of Cardiology, The First Affiliated Hospital of Guangxi Medical University, Nanning, China; 3 Department of Orthopedic Trauma, Zhuhai People’s Hospital (Zhuhai Hospital Affiliated with Jinan University), Zhuhai, China; 4 Department of Hypertension and Vascular Disease, The First Affiliated Hospital, Sun Yat-sen University, Guangzhou, China; 5 Zhuhai Precision Medical Center, Zhuhai People’s Hospital (Zhuhai Hospital Affiliated with Jinan University), Zhuhai, China; 6 Wards of Cadres, Zhuhai People’s Hospital (Zhuhai Hospital Affiliated with Jinan University), Zhuhai, China

**Keywords:** Cytokines, Long non-coding RNAs

## Abstract

Exosomes are nanosized bilayer membrane vesicles that may mediate intercellular communication by transporting bioactive molecules, including noncoding RNAs, mRNAs, and proteins. Research has shown that exosomes play an important role in acute myocardial infarction (AMI), but the function and regulation of exosomal long noncoding RNAs (lncRNAs) in AMI are unclear. Thus, RNA sequencing (RNA-Seq) was conducted to investigate the exosomal lncRNA transcriptome from MI patients and identified 65 differentially expressed lncRNAs between the MI and control groups. HCG15, one of the differentially expressed lncRNAs, was verified to have the highest correlation with cTnT by qRT-PCR, and it also contributed to the diagnosis of AMI by receiver operating characteristic (ROC) analysis. Upregulation of HCG15 expression facilitated cardiomyocyte apoptosis and inflammatory cytokine production and inhibited cell proliferation. We also confirmed that HCG15 was mainly wrapped in exosomes from AC16 cardiomyocytes under hypoxia, which contributed to cardiomyocyte apoptosis, the release of inflammatory factors, and inhibition of cell proliferation via the activation of the NF-κB/p65 and p38 pathways, whereas suppressing the expression of HCG15exerted opposite effects, In addition, Overexpression of HCG15 aggravated cardiac IR injury in C57BL/6J mice. This study not only helps elucidate exosomal lncRNA function in AMI pathogenesis but also contributes to the development of novel therapeutic strategies.

## Introduction

Acute myocardial infarction (AMI), which is mainly caused by coronary artery occlusion and myocardial ischemia, remains a serious cardiovascular disease with high morbidity and mortality [1, 2]. AMI can lead to congestive heart failure and malignant arrhythmia, which seriously threaten human health [3]. The sensitive and early diagnosis of AMI has become a research focus, as it will provide benefits for the treatment of AMI and improve the survival and cure rates of patients [4]. In the clinic, cardiac troponins are the most frequently used markers in the diagnosis of AMI. However, several conditions other than AMI, such as myocarditis and malignant tumors, may lead to elevated levels of cardiac troponins. Thus, exploration of specific markers in the early clinical diagnosis of AMI and clarification of the potential mechanisms are important.

Long noncoding RNAs (lncRNAs) are a novel group of noncoding RNAs with >200 nucleotides. Although lncRNAs cannot encode proteins, they have been proven to be involved in cell growth, differentiation, proliferation, and apoptosis [3]. LncRNAs regulate gene expression by cooperating with transcription factors (TFs) or remodeling complexes and can maintain protein stability [5]. Recently, numerous studies have reported that circulating lncRNAs, such as LIPCAR [6], UCA1 [4], NRON, and MHRT [7], play an important role in the pathogenesis of heart failure. LncRNA Meg3 could promote myocardial cell apoptosis, while knocking down Meg3 significantly improved cardiac function in mice with MI [8]. The microarray dataset GSE66360 showed that lncRNA SLC8A1-AS1 protected against MI by activating the cGMP-PKG pathway in mice with MI [9]. Enhanced expression of lncRNA ECRAR could substantially stimulate myocardial regeneration and induce recovery of cardiac function after MI, suggesting that ECRAR may be an effective therapeutic target for heart failure [10]. Therefore, systemic exploration of the differentially expressed lncRNAs in AMI, screening of potential functional lncRNAs and investigation of their roles and mechanisms in AMI are urgently needed.

Exosomes are nanosized (30–100 nm in diameter) membrane-wrapped vesicles that may be involved in intercellular communication by transporting proteins and RNA molecules [11]. Emerging evidence has shown that exosomes from stem cells or cardiac progenitor cells facilitate cardiac repair and ameliorate AMI [12–14]. In particular, exosome-mediated miRNA and mRNA transfer between cardiomyocytes affects cardiac physiology and pathological state [2, 15–17]. However, the function and regulation of exosomal lncRNAs in AMI are poorly understood.

In this study, RNA sequencing (RNA-Seq) was performed to investigate the exosomal lncRNA transcriptome from AMI patients, and an anoxic model of AC16 cardiomyocytes was established. We confirmed that lncRNA HLA complex group 15 (HCG15) expression was upregulated in exosomes from AMI patients and hypoxia-induced cells, which resulted in cardiomyocyte apoptosis, the release of inflammatory cytokines and inhibition of cell proliferation. These findings may contribute to the development of novel therapeutic strategies.

## Materials and methods

### Patients and samples

Blood samples of AMI patients were collected within 12 h after the onset of chest pain. The control samples were obtained on the morning after admission in a fasting state. Serum was separated and stored at −80 °C within half an hour.

### Isolation and identification of exosomes

Exosomes from serum were isolated using ExoQuick precipitation solution (SBI, USA) according to the manufacturer’s protocol. Briefly, 1 ml of serum was filtered with a 0.22-μm pore filter. Then, cell-free serum was mixed with ExoQuick precipitation solution. After the mixture was incubated for 30 min at 4 °C and centrifuged at 1500 × *g* for 30 min at 4 °C, the obtained pellets were resuspended in PBS and stored at −80 °C until use.

Conditioned medium (10 ml) was collected from normoxic and hypoxic AC16 cells cultured in DMEM (Gibco, USA) with 10% exosome-depleted fetal bovine serum (FBS) (Gibco, USA) for 24 h and centrifuged at 3000 × *g* for 15 min to remove cells and debris. After filtered with a 0.2-μm pore filter, the supernatant was further concentrated by centrifugation at 3000 × *g* for 20 min at 4 °C in an ultrafiltration tube (Amicon Ultra-15, Millipore, USA). The concentrated samples were mixed with ExoQuick-TC precipitation solution by vortexing, incubated overnight, and finally centrifuged at 1500 × *g* for 30 min at 4 °C to isolate the pellets. The pellets were resuspended in PBS and stored at −80 °C until use.

Exosome pellets were resuspended in 2% paraformaldehyde in PBS, pH 7.4, and the resuspended exosome pellets were dropped on formvar-carbon-coated 200-mesh copper electron microsocpy grids. After staining with uranyl acetate (1.75%), the exosome morphology was observed with transmission electron microscopy (TEM, HT7700, Hitachi, Japan). Exosome sizes were determined by the NanoSight NS300 system (NanoSight Technology, Malvern, UK). The expression levels of exosome-specific biomarkers (CD63, CD9, and TSG101) and the negative indicator calnexin were detected by western blot analysis. Exosomes were quantified by mass concentration with a NanoDrop 2000 Spectrophotometer (Thermo, Waltham, MA, USA).

### RNA-Seq analysis

Total RNA of exosomes (20 μg) from six AMI patients and six healthy controls (clinical characteristics shown in Supplementary Table 1) was extracted using TRIzol reagent (Life Technologies, USA) following the instructions. The preparation of whole transcriptome libraries and deep sequencing were performed by Novogene Bioinformatics Technology Corporation (Shanghai, China). Ribosomal RNA was eliminated, and strand-specific sequencing libraries were constructed according to the protocol. RNA-Seq was conducted on an Illumina HiSeq 2000 sequencer (Illumina, San Diego, USA), and 150 bp reads were produced conforming to Illumina’s instructions.

After low-quality data were filtered, the clean reads were aligned to the human reference genome GRCh37/hg19 by the HISAT2 program. Then, transcripts were spliced and assembled using StringTie software. Information about lncRNAs was annotated based on GENCODE [18]. The fragment per kilobase of transcript per million mapped reads (FPKM) values were used to evaluate the expression abundance for each transcriptional region, which was calculated by StringTie software. Differential expression of RNAs with a *p* value < 0.05 and fold change (FC) ≥ 2 was identified by DESeq2 software.

### Cell culture, hypoxic treatment, and transfection

The human cardiomyocyte cell line AC16 was obtained from the BeNa Culture Collection (Beijing, China) and cultured in DMEM (Gibco, USA) supplemented with 10% FBS (Gibco, USA). Normoxic cells were cultured in a humid atmosphere of 37 °C, 95% air, and 5% CO_2_. For simulation of the state of ischemic injury in vitro, cells were pre-exposed to 95% N_2_ and 5% CO_2_ for 2 h and placed in a hypoxic incubator (Sanyo, Japan) with 1% O_2_ for different durations.

HCG15 overexpression plasmids were constructed by cloning the sequences of HCG15 (synthesized by General Biosystems, Chuzhou, China) into the pcDNA3.1 vector. SiRNAs targeting lncRNA HCG15 were transfected into AC16 cells, and a nontargeting siRNA (scrambled) was used as the negative control. Cells were seeded in six-well plates at 1 × 10^6^ cells per well. HCG15 overexpression plasmids or siRNAs (siRNA-1, siRNA-2, and siRNA-3) were transfected into AC16 cells with Lipofectamine 2000 (Invitrogen, USA) in Opti-Minimal Essential Medium (Gibco, USA). Experiments were conducted 48 h after transfection to assess the silencing effect. The sequences of siRNA-1, siRNA-2, siRNA-3 and scramble were following: HCG15 siRNA-1: sense: GCU CGU UAC UGC UCA GAA UTT, antisense: AUU CUG AGC AGU AAC GAG CTT; HCG15 siRNA-2: sense: GAG CUC UAU GCC AUC CUG CTT, antisense: GCA GGA UGG CAU AGA GCU CTT; HCG15 siRNA-3: sense: GAA GGU UAA AGC UGU CAA ATT, antisense: UUU GAC AGC UUU AAC CUU CTT; scramble sense: UUC UCC GAA CGU GUC AC GUTT, and antisense: ACG UGA CAC GUU CGG AGA ATT.

### Labeling and uptake of exosomes

Exosomes were extracted from the culture medium of AC16 cells under hypoxia for 8 h and labeled with PKH67 dye (Sigma-Aldrich, USA) at room temperature for 5 min. Then, exosome-depleted bovine serum albumin was added to labeling reactions. Subsequently, exosomes were washed three times with an ultrafiltration tube (Amicon Ultra-15, Millipore, USA) to remove unbound dyes. Finally, AC16 cells (2 × 10^5^) were incubated with labeled exosomes. Normoxic AC16 cells that assimilated exosomes from hypoxic AC16 cells were monitored using confocal microscopy (LSM880, Zeiss, Germany).

### qRT-PCR analysis

Total RNA was isolated with TRIzol (Life Technologies, USA), and DNA contamination was eliminated with DNase I. The purified RNA was reverse transcribed with a First Strand cDNA Synthesis Kit (Thermo Fisher, MA, USA). Real-time PCR was conducted using SYBR Premix (Tiangen, Beijing, China) and a LightCycler 480 system (Roche, Basel, Switzerland). β-actin was used as the reference gene for normalization. Primers used for lncRNA HCG15 and β-actin were as follows: HCG15, 5′-CGC GGG TCA CCT TCT GAA TTT-3′ (forward), 5′-AAA GAG CGC AGT CCT TGC TG-3′ (reverse); β-actin, 5′-TTC CTT CCT GGG CAT GGA GTC-3′ (forward), 5′-TCT TCA TTG TGC TGG GTG CC-3′ (reverse). The exosomal RNA levels were normalized to the exogenous λ polyA RNA level (TaKaRa, Japan) for qRT-PCR. All reactions were performed in triplicate. Amplification products were quantified using the 2^−△△Ct^ method.

### Western blot analysis

Proteins were extracted with SDS lysis buffer (50 mM Tris-HCl, pH 6.8, 2.2% SDS, 5.5% glycerol and 1 mM PMSF). AC16 cells and isolated exosomes were washed with PBS and resuspended in SDS lysis buffer followed by centrifugation at 4 °C and 16,000 × *g* for 30 min and supernatant collection. Protein was quantified using the BCA Protein Assay Kit (Thermo Fisher, MA, USA). Equal amounts of proteins were subjected to 10% SDS-PAGE and transferred onto a polyvinylidene difluoride (PVDF) membrane (Millipore, USA). The membranes were blocked with 5% nonfat dried milk in PBS and incubated with primary antibodies, including anti-CD63 antibody, anti-TSG101 antibody (CST, Boston, MA, USA), anti-CD9 antibody (Abcam, Cambridge, UK), anti-calnexin antibody (Abcam, Cambridge, UK), anti-JNK antibody, anti-phospho-SAPK/JNK (Thr183/Tyr185) antibody, anti-NF-κB p65 antibody, anti-phospho-NF-κB p65 (Ser536) antibody, anti-p44/42 MAPK (Erk1/2) antibody, anti-phospho-p44/42 MAPK (Erk1/2) (Thr202/Tyr204) antibody, anti-p38 MAPK antibody, anti-phospho-p38 MAPK (Thr180/Tyr182) antibody, and anti-GAPDH antibody (CST, Boston, MA, USA), at 4 °C overnight. Then, the samples were incubated with HRP-conjugated goat anti-rabbit or mouse IgG antibody (Abcam, Cambridge, UK) for 2 h. The blot signal was detected with enhanced chemiluminescence reagent (Thermo Fisher, MA, USA). Image-Pro Plus software 6.0 was used for densitometric analysis.

### Cell viability analysis

AC16 cell viability was detected by MTT (3-[4,5-dimethylthiazyol-2yl]-2,5-diphenyltetrazolium bromide, Sigma) assays. First, 5 × 10^3^ cells/well were seeded into 96-well plates. After 24 h of culture, 20 µl of MTT solution was added to each well and maintained at 37 °C for 4 h. After the medium was removed, 150 µl of dimethylsulfoxide (DMSO) was added to each well and incubated at 37 °C for 10 min. Subsequently, the absorbance was measured at 570 nm using a Multiskan MK microplate reader (Thermo Scientific, USA).

### Detection of cell apoptosis

The terminal deoxynucleotidyl transferase-mediated dUTP nick end labeling (TUNEL) (Roche, Mannheim, Germany) method was applied to detect apoptosis. Briefly, cells in each treated group were immobilized with 4% paraformaldehyde at room temperature. Subsequently, the cells were washed twice with PBS and incubated with buffer containing 0.1% Triton X-100 on ice for 2 min. Next, the cells were washed and sealed with 3% BSA. Finally, the slides were incubated with 50 μl of freshly prepared TUNEL reaction mixture for 1 h at 37 °C in a humidified chamber. Fluorescent images were acquired with fluorescence microscopy (AE31, Motic, Xiamen, China).

Flow cytometry was also used to detect cell apoptosis. After specific treatment, the cells were digested with 0.25% trypsin and collected by centrifugation at 2000 rpm for 5 min. An Annexin V-FITC/PI Apoptosis Detection Kit (Bioleng, CA, USA) was used for apoptotic detection. Cells were suspended in 500 μl of binding buffer, and then, 5 ml of annexin V-FITC was added and fully mixed. Propidium iodide (5 μl) was added and incubated in the dark at room temperature for 10 min. Finally, the cells were analyzed within 1 h by flow cytometry (CytoFlex, Beckman, CA, USA).

### Enzyme-linked immunosorbent assay

The inflammatory cytokines IL-6, IL-1β, and TNF-α released from the supernatant were measured by enzyme-linked immunosorbent assay kits (ELISA) (Immunoway, TX, USA). Optical density (OD) values were measured at 450 nm by a Multiskan MK microplate reader (Thermo Scientific, USA). The OD values for each sample were compared with standard curves to quantify the amount of protein in the original samples.

### Detection of the level of cTnT

cTnT was detected by a Cobas E601 chemiluminescence analyzer (Roche, Basel, Switzerland) and supporting reagents. The detection range of reagents was 0.003–10 ng/ml.

### Mouse model of myocardial ischemia–reperfusion injury

The protocol was performed following the guidelines approved by the Institutional Animal Care and Use Committee of Southern Medical University. All animal care and experimental protocols were in compliance with the National Institutes of Health guidelines for the care and use of laboratory animals. Male C57BL/6J mice (8–10 weeks) were used in this study. Mice with normal lipid metabolism were provided by the Experimental Animal Center of Southern Medical University. All mice used in the study were fed normal mouse food. The mice were kept under pathogen-free conditions and maintained at a standard temperature and humidity.

Mice were anesthetized with an intraperitoneal injection of 100 mg/kg ketamine (Ketathesia) and 10 mg/kg xylazine and intubated for assisted respiration using a small animal ventilator (Harvard Apparatus, Natick, MA). The neck and chest of the mice were cleaned and disinfected with 75% ethanol. The trachea was intubated from the mouth of the mice. After successful intubation, the cannula was fixed, and the ventilator was quickly connected to assist breathing. After left lateral thoracotomy, the pericardium was dissected, and an 8-0 surgical suture was carefully passed underneath the left anterior descending coronary artery (LAD) at a position 1–2.5 mm from the tip of the left auricle. The suture was cut at the needle site, and both ends were threaded through a 1 mm section of PE-10 tubing, forming a loose snare around the LAD. Ischemia for 45 min and reperfusion were produced by loosening the silk thread. Cardiac ischemia was confirmed by a pale area below the suture or ST-T elevation shown in the ECG that gradually became cyanotic, while reperfusion was characterized by the rapid disappearance of cyanosis followed by vascular blush. The sham group underwent the same surgical procedures but with no coronary artery ligation.

### Quantification of infarct size

At 24 h post-reperfusion, mice were reanesthetized and reintubated, and the LAD was reoccluded by ligating the suture in the same position as for the original infarction. Animals were then killed, and 1 ml of 1% Evans Blue dye (Sigma) was infused intravenously (i.v.) to delineate the area at risk (AAR, corresponding to the myocardium lacking blood flow). The left ventricle (LV) was isolated and cut into transverse slices (5–7 1-mm slices per LV), and both sides were imaged. For delineation of the infarcted (necrotic) myocardium, slices were incubated in triphenyltetrazolium chloride (TTC, Sigma) at 37 °C for 15 min. The slices were then rephotographed and weighed, and regions negative for Evans Blue staining (AAR) and TTC (infarcted myocardium) were quantified with ImageJ. Percentage values for AAR and infarcted myocardium were independently mass corrected for each slice.

### Echocardiography

Four weeks after surgery, transthoracic echocardiography was performed to evaluate cardiac function. Echocardiography was performed as previously described [19]. Echocardiographic analysis using a Vevo2100 digital imaging system (Visual Sonics) was performed under 1% isoflurane, with midventricular M and B mode measurements acquired in the parasternal short-axis view at the level of the papillary muscles. Once the mice were acclimated to the procedures, images were stored in digital format on a magnetic optical disk for review and analysis. M-mode tracings were recorded through the anterior and posterior LV walls at the papillary muscle level in the same long axis views to measure left ventricular end-diastolic diameter (LVEDD) and LV end-systolic diameter (LVESD), as well as the interventricular septum (IVS) and posterior wall (PW) dimensions. The left ventricular ejection fraction (EF) was calculated by the cubic method: LVEF (%) = ((LVIDd)^3^–(LVIDs)^3^)/(LVIDd)^3^ × 100, and the left ventricular FS was calculated by LVFS (%) = (LVIDd–LVIDs)/LVIDd × 100. The data were averaged from five cardiac cycles.

### Masson staining

The mice were killed at 4 weeks after ischemia–reperfusion (IR). The hearts were collected and fixed in 4% formaldehyde solution for 24–48 h. Then, the hearts were dehydrated and paraffin-embedded. Next, 5-µm-thick slices were cut for Masson’s trichrome staining to visualize fibrosis. Images were captured by microscopy and analyzed by ImageJ.

### Overexpression of lncRNA HCG15

For in vivo infection, pAAV9-CMV-ZsGreen-HCG15 (Oe-HCG15) or control (scramble) virus particles (1 × 10^11^ viral genomes/ml) were administered by direct injection with a 30-gauge syringe needle into the free wall, apex, and sidewall of the LV (three sites, 8 μl/site) in 8-week-old mice. Four weeks later, the mice were subjected to IR or sham surgery.

### Intervention with the NF-κB antagonist (SN50) or p38 MAPK antagonist (ralimetinib (LY2228820) dimesylate) in IR mice

Male C57BL/6J mice were injected via tail veil with Oe-HCG15 virus. At 25 days after infection, we treated mice with daily intraperitoneal injection of SN50 (10 μg/kg per day) 3 days before IR model establishment. The treatment was last for 4 weeks.

To block p38 MAPK signaling pathway, Oe-HCG15 mice were given ralimetinib (LY2228820) dimesylate (20 mg/kg) dissolved in saline by the oral administration 3 days before model establishment and last for 4 weeks.

### Statistical analysis

SPSS Statistics 20.0 was used to analyze the data. The data are presented as the mean ± SD. Each assay was performed at least three times. Comparisons between groups were performed using Student’s *t* test for two groups of data and one-way ANOVA followed by Tukey’s multiple comparison test for multiple groups of data. *p* < 0.05 was considered statistically significant.

## Results

### Identification of differentially expressed exosomal lncRNAs from the serum of AMI patients

**Fig. 1:**
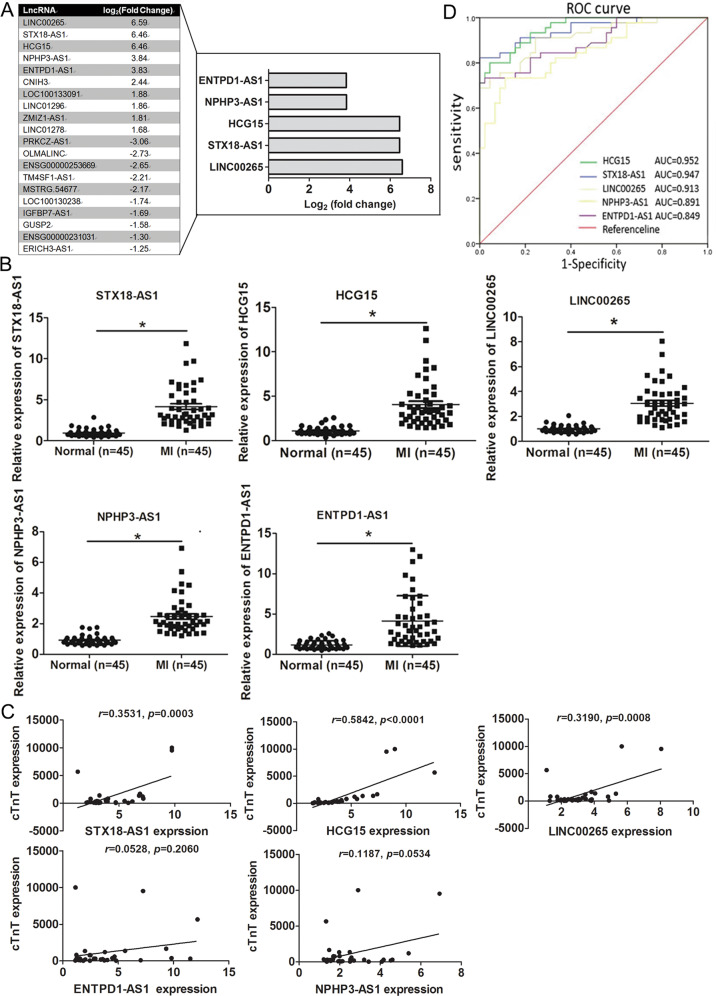
Identification of the differentially expressed exosomal lncRNAs from the serum of AMI patients and healthy controls. **A** The top 10 lncRNAs with upregulated and downregulated expression according to the fold change value in AMI patients compared with controls and the top 5 lncRNAs with upregulated expression are shown in the bar chart. **B** Validation of the top 5 lncRNAs with upregulated expression in study participants by qRT-PCR. **C** The levels of 5 lncRNAs in exosomes derived from MI patients’ serum were positively correlated with the cTnT concentration of AMI patients. **D** ROC curve analysis of the clinical diagnostic value of these 5 lncRNAs in MI. *p value < 0.05.

The expression profiles of exosomal lncRNAs from six AMI patients and six matched healthy controls were determined by RNA-Seq, and their clinical characteristics are shown in Supplementary Table 1. First, exosomes were extracted and identified by TEM and dynamic light scattering; most of the exosomes were ~100 nm in diameter (Fig. S1A, B). Second, total RNA isolated from exosomes was used for the preparation and sequencing of the cDNA library. As shown by the volcano plot (Fig. S1C), a total of 65 differentially expressed lncRNAs were identified between the AMI patients and the healthy controls based on the following criteria: log_2_ (fold change) > 1 and padj-value < 0.05. Twenty-nine lncRNAs with upregulated expression and 36 with downregulated expression were identified in the AMI group, and the top ten lncRNAs with upregulated and downregulated expression are shown in Fig. 1A. To validate the RNA-Seq results, we assessed the top five lncRNAs with upregulated expression (STX18-AS1, HCG15, LINC00265, NPH3-AS1, and ENTPD1-AS1; Fig. 1A and Supplementary Table 2) through qRT-PCR analyses of 45 AMI patients and 45 healthy volunteers. The clinical characteristics of the cohort are displayed in Supplementary Table 3. There were obvious differences in sex, blood pressure, creatinine, LDL, and cTnT expression between the AMI patients and the healthy controls. All five lncRNAs showed significantly upregulated expression in the AMI group (Fig. 1B), which was consistent with the RNA-Seq data. Subsequently, we examined the correlation between lncRNAs and cTnT. The results showed a positive correlation between the expression of these 5 lncRNAs and the cTnT concentration, and the expression of HCG15 had the highest positive correlation with cTnT (Fig. 1C). In addition, we performed receiver operating characteristic (ROC) analysis to assess the clinical diagnostic value of the five lncRNAs in MI. The area under the receiver operating characteristic curve (AUC) values of the five lncRNAs ranged from 0.849 to 0.952 (Fig. 1D), and the AUC value of lncRNA HCG15 was the highest. Thus, we speculated that HCG15 might play an important role in MI, and we focused on HCG15 in further studies.

### LncRNA HCG15-induced cardiomyocyte apoptosis and the production of inflammatory cytokines

**Fig. 2:**
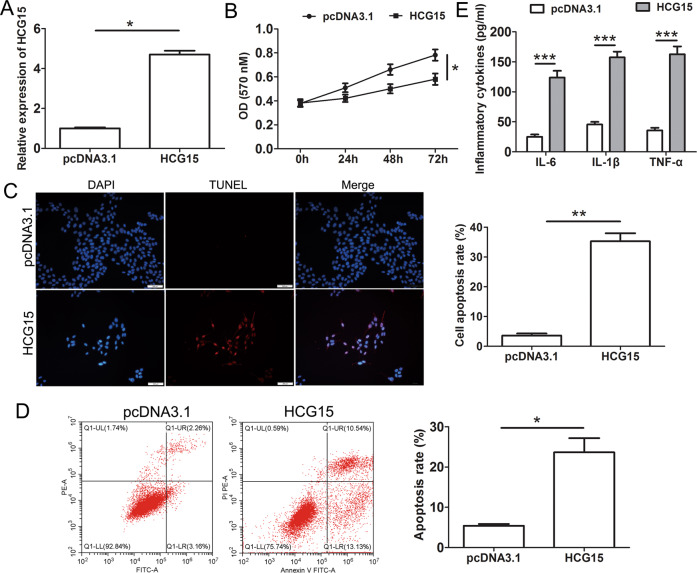
LncRNA HCG15-induced cardiomyocytes apoptosis and the production of inflammatory cytokines. **A** qRT-PCR was performed to examined HCG15 expression in AC16 cells after transfection of the HCG15 overexpression plasmid. **B** MTT assays detected the proliferation of AC16 cells after transfection of the HCG15 overexpression plasmid. TUNEL staining (**C**) and flow cytometry (**D**) were performed to measure the apoptosis of AC16 cells after transfection with the HCG15 overexpression plasmid. **E** The production of inflammatory cytokines of AC16 cells transfected with the HCG15 overexpression plasmid was measured by ELISAs. *p value < 0.05, **p value < 0.001, ***p value < 0.001. Each assay was performed in triplicate.

To investigate the biological role of HCG15 in cardiomyocytes, we transfected a HCG15 overexpression plasmid or control vector into AC16 cells. qRT-PCR confirmed that HCG15 expression was significantly elevated in the AC16 cells transfected with the HCG15-overexpressing plasmids (Fig. 2A). Proliferation was suppressed after HCG15 overexpression, as shown by MTT (5 mg/ml, Sigma) assays (Fig. 2B). TUNEL staining (Fig. 2C) and flow cytometry (Fig. 2D) showed that cell apoptosis was increased after HCG15 overexpression. In addition, the levels of inflammatory cytokines, such as IL-6, IL-1β, and TNF-α, were strongly increased when AC16 cells were transfected with the HCG15 overexpression vector (Fig. 2E).

### HCG15 was transferred to cardiomyocytes via exosomes after hypoxia

**Fig. 3:**
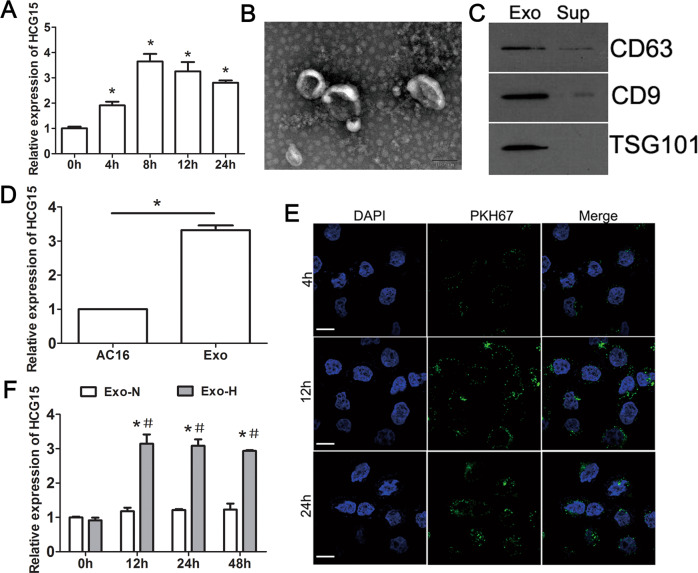
HCG15 carried by exosomes was transferred to cardiomyocytes after hypoxia. **A** qRT-PCR detected the expression of HCG15 in hypoxic AC16 cells. **B** Exosomes isolated from hypoxic AC16 cells are shown by TEM. **C** Western blotting was used to detect the expression of biomarkers in the exosome (Exo) and supernatant fractions (Sup) of the medium from AC16 cells after 8 h of hypoxia. **D** qRT-PCR was used to detect the level of HCG15 in exosomes and their producers (AC16 cells) after hypoxic treatment. **E** Confocal microscopy showed that PKH67-labeled exosomes from hypoxic AC16 cells were taken up by AC16 cells at different times. The nuclei were stained with DAPI; green: PKH67 (the magnification was × 400). **F** Expression of HCG15 in AC16 cells after incubation with Exo-N and Exo-H at different times was detected by qRT-PCR. *p value < 0.05 vs 0 h, #*p* value < 0.05 vs Exo-N. Each assay was performed in triplicate.

To explore how HCG15 functions and to simulate hypoxic conditions in vitro, we analyzed hypoxic AC16 cells. As shown in Fig. 3A, the level of HCG15 was significantly upregulated after hypoxia and peaked at 8 h. Exosomes were extracted from the culture media of AC16 cells under normal and hypoxic conditions and then identified by TEM (Fig. 3B). The exosomal markers CD63, CD9, and TSG101, but not calnexin, were detected by western blotting (Figs. 3C and S1D). Moreover, HCG15 was enriched in exosomes, as the level was over three times higher than that of producer cells (Fig. 3D). To identify whether exosomes (exosomes derived from hypoxic cells were named Exo-H and exosomes derived from normoxic cells were named Exo-N) were endocytosed by normoxic cells, we incubated AC16 cells with Exo-H labeled with PKH67 for different durations. After 4 h, 12 h, and 24 h of incubation, green signals were detected in the normoxic AC16 cells (Fig. 3E). This finding indicated that hypoxic exosomes could be effectively taken up by AC16 cells. To investigate whether exosomes could transfer HCG15 to receptor cells and whether HCG15 was released directly in exosomes, we incubated AC16 cells with Exo-H or Exo-N. The expression of HCG15 in the AC16 cells incubated with Exo-H was significantly increased compared to that of the cells incubated with Exo-N (Fig. 3F). These findings suggested that exosome-mediated HCG15 may be involved in cardiomyocyte apoptosis.

### LncRNA HCG15 in Exo-H induced cardiomyocyte apoptosis and the production of inflammatory cytokines

**Fig. 4:**
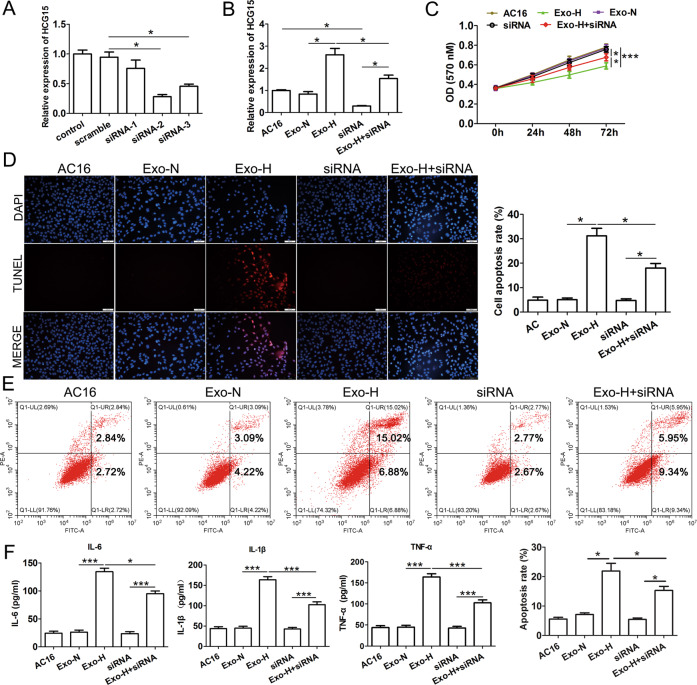
LncRNA HCG15 in exosomes released from hypoxic cells contributed to cardiomyocyte apoptosis and the production of inflammatory cytokines. **A** qRT-PCR was performed to determine the expression of HCG15 in AC16 cells after transfection with HCG15 siRNAs. **B** qRT-PCR was performed to detect the expression of HCG15 in AC16 cells after transfection with HCG15 siRNA and subsequent incubation with Exo-H. **C** MTT assays detected the proliferation of AC16 cells after transfection with HCG15 siRNA and subsequent incubation with Exo-H. TUNEL staining (**D**) and flow cytometry (**E**) were performed to measure the apoptosis of AC16 cells after the indicated treatment. **F** ELISAs were used to measure the production of inflammatory cytokines in AC16 cells after the indicated treatment. *p value < 0.05, ***p value < 0.001. Each assay was performed in triplicate.

To verify whether HCG15 in Exo-H cells was involved in myocardial cell injury, we transfected three different siRNAs targeting lncRNA HCG15 (named siRNA-1, 2, and 3) into AC16 cells. Only siRNA-2 and siRNA-3 effectively interfered with the expression of endogenous HCG15 (Fig. 4A), and siRNA-2 showed the best interference effect, so siRNA-2 was selected for the following assays. The expression of lncRNA HCG15 in the AC16 cells incubated with Exo-H increased significantly and was attenuated by HCG15 siRNA transfection (Fig. 4B). MTT analysis showed that cell proliferation was significantly inhibited after incubation with Exo-H, but this inhibition was significantly attenuated when HCG15 siRNA was transfected after incubation with Exo-H (Fig. 4C). In particular, when cells were incubated with Exo-H, cell apoptosis was increased significantly, but after incubation with Exo-H and transfection with HCG15 siRNA, cell apoptosis was strongly alleviated, as shown by TUNEL staining (Fig. 4D) and flow cytometry (Fig. 4E). In addition, inflammatory factors such as IL-6, IL-1β, and TNF-α from AC16 cells were examined by ELISAs. After incubation with Exo-H, the production of inflammatory cytokines was substantially increased, but their expression levels were reduced when cells were transfected with HCG15 siRNA (Fig. 4F). These results suggested that reducing exosomal lncRNA HCG15 levels released from cells under hypoxia may attenuate myocardial injury.

### Mechanisms of lncRNA HCG15-induced cardiomyocyte injury in exosomes released by hypoxic cells

**Fig. 5:**
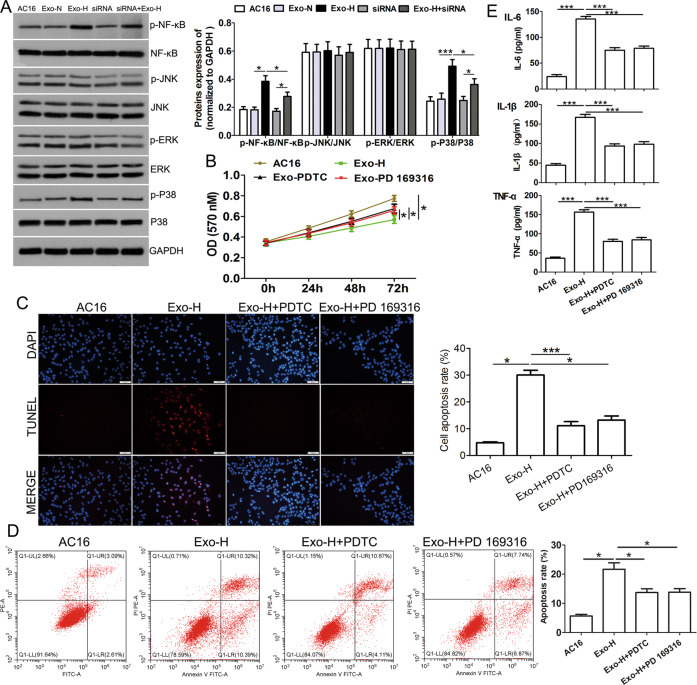
Exosomal HCG15 released from hypoxic cells promoted cardiomyocyte apoptosis and the production of inflammatory cytokines via the NF-κB/p65 and p38 pathways. **A** Western blotting was used to analyze the expression of NF-κB/p65, JNK, ERK, and p38 signaling pathway molecules in AC16 cells after the indicated treatments. AC16 cells were incubated with Exo-H and subsequently treated with PDTC (0.1 mg/ml) or PD169316 (10 μmol/l), and MTT assays (**B**) were used to detect cell proliferation. TUNEL staining (**C**) and flow cytometry (**D**) were performed to measure cell apoptosis, and the magnification was 200 ×. **E** ELISA was used to measure the level of inflammatory cytokines. *p value < 0.05, ***p value < 0.001. Each assay was performed in triplicate.

To elucidate the possible molecular mechanisms of lncRNA HCG15 in exosomes released from hypoxic cells that mediate cardiomyocyte injury, we assessed components of the NF-κB and MAPK signaling pathways, such as p38 MAPK, JNK1/2, and ERK1/2, by western blot analysis. The results showed that the expression of phosphorylated NF-κB p65 and p38 was upregulated when AC16 cells were incubated with Exo-H (Fig. 5A). This upregulation was antagonized when cells were transfected with HCG15 siRNA after incubation with Exo-H, while other signaling molecules did not show obvious changes (Fig. 5A), which indicated that the NF-κb/p65 and p38 pathways were activated during this process. Furthermore, we blocked the NF-κB/p65 and p38 pathways with the specific signaling repressors PDTC and PD169316, respectively, and found that apoptosis and inhibition of cell growth were antagonized (Fig. 5B–D). Moreover, after the NF-κB/p65 and p38 pathways were blocked, the elevated levels of inflammatory cytokines induced by Exo-H were strongly attenuated (Fig. 5E). Thus, lncRNA HCG15 in exosomes released from hypoxic cells may mediate cardiomyocyte injury through the NF-κB/p65 and p38 pathways.

### Overexpression of lncRNA HCG15 aggravated cardiac IR injury

**Fig. 6:**
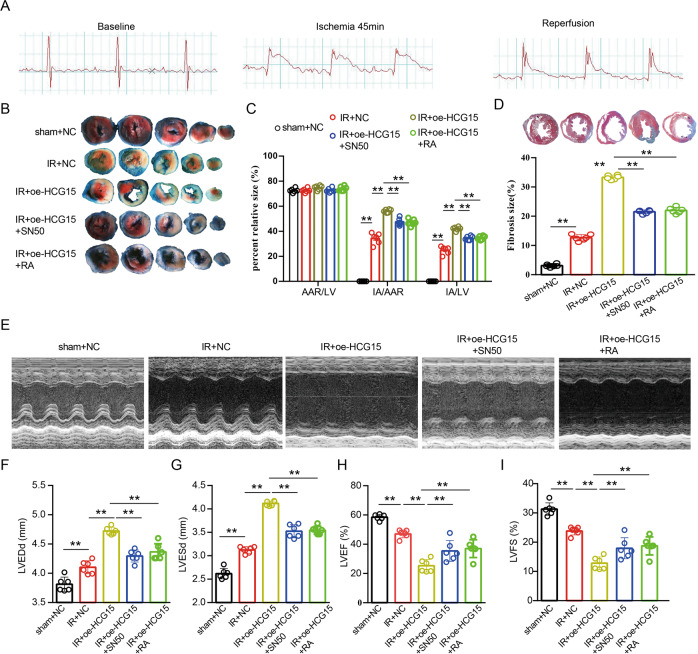
Overexpression of HCG15 aggravated cardiac IR injury. **A** ECG of mice in response to cardiac IR. **B** Representative images of Evans blue and triphenyl tetrazolium chloride (TTC) double-stained myocardial sections from mice at 4 weeks after cardiac IR; scale bar = 2 mm. **C** Myocardial IS in the indicated groups at 4 weeks after surgery; *n* = 5 per group, **p < 0.05. **D** Representative images of Masson staining of ventricular sections from mice at 4 weeks after cardiac IR and quantitative analyses of infarct size; scale bar = 1 mm; *n* = 5 per group, **p < 0.05. **E**–**I** Representative images of M-mode echocardiography (**E**) and relative indices of LVEDD (**F**), LVESD (**G**), LVEF (**H**), and LVFS (**I**) in mice at 4 weeks after cardiac IR, *n* = 5 per group, **p < 0.05. NF-κB antagonist (SN50); p38 MAPK antagonist (ralimetinib (LY2228820) dimesylate); (RA: ralimetinib (LY2228820) dimesylate).

To investigate the role of HCG15 in cardiac IR injury, we subjected male C57BL/6J mice to IR after transfection with oe-HCG15 or oe-HCG15 together with NF-κB-specific inhibitors or oe-HCG15 together with p38 pathway-specific inhibitors. ST segments were significantly elevated after LCA ligation for 45 min and returned to baseline after reperfusion (Fig. 6A), indicating successful modeling. Histological analyses were performed 4 weeks after surgery. Compared with that in the sham group, the ischemia size (IS) in the IR group was substantially inecreased. The IR-induced mice treated with oe-HCG15 showed a significantly wider IS than the control IR-induced mice. Furthermore, we found that inhibition of NF-κB/p65 or p38 pathway partially reversed the effect of lncRNA HCG15 overexpression (Fig. 6B, C). Correspondingly, the oe-HCG15-treated IR-induced mice had a larger infarct scar size, while blocking the NF-κB/p65 or p38 pathway with their specific inhibitors decreased the infarct scar size in the oe-HCG15-treated IR-induced mice (Fig. 6D). Echocardiography showed that the oe-HCG15-treated IR-induced mice had a higher LVEDD and LVESD and a lower ejection fraction (LVEF) and fractional shortening (LVFS). Compared with those of the oe-HCG15-treated IR-induced mice, the cardiac functions were preserved in the oe-HCG15-treated IR-induced mice treated with the NF-κB/p65 and p38 pathway inhibitors (Fig. 6E–I).

These findings indicated that overexpression of HCG15 aggravates cardiac IR injury, which was alleviated by blocking the NF-κB and p38 pathways.

## Discussion

In this study, we identified lncRNA HCG15 as a novel diagnostic marker that was one of the top five lncRNAs with upregulated expression between AMI patients and healthy controls and had the highest correlation with cTnT. We discovered that HCG15 was enriched in exosomes derived from hypoxic AC16 cells and that reducing lncRNA HCG15 levels could decrease cell apoptosis, reduce the release of inflammatory factors and promote cell proliferation in cardiomyocytes. Overexpression of HCG15 aggravated cardiac IR injury in C57BL/6J mice. To the best of our knowledge, this is the first study to reveal the function of HCG15 in AMI.

Exosomes, which are small bilayer membrane vesicles, can be extracted from most body fluids, such as urine, saliva and plasma [20]. Lipids, proteins, RNAs (microRNAs, mRNAs and ncRNAs), and DNA are the biological cargos of exosomes secreted by most human cells [21]. Increasing evidence has demonstrated that exosomes carrying bioactive molecules can be transferred to receptor cells, which affects various biological processes, including inflammation, metabolic autoimmunity, tumor development, and cardiovascular diseases [22]. Notably, exosomes play an important role in mediating intercellular communication. Since Valadi et al. first reported that exosomes mediated miRNA transfer [11], accumulating evidence has suggested that proteins and miRNAs in exosomes released by cardiomyocytes can be transferred to myocardial endothelial cells and affect their functions [23, 24]. Exosomes secreted by hypoxic cardiomyocytes harboring TNF-α could aggravate the apoptosis of receptor cardiomyocytes [25]. According to Yang’s report, miR-30a is rich in exosomes derived from cardiomyocytes under hypoxic stimulation. Inhibiting the release of miR-30a or exosomes led to maintenance of autophagy and reduced apoptosis in cardiomyocytes after hypoxia [26]. Furthermore, exosomal miRNA-194 led to cardiac injury and mitochondrial dysfunction in obese mice [27]. In our research, we isolated exosomes from hypoxic cardiomyocytes and identified their morphology and biomarkers. Moreover, we found that exosome-mediated intercellular communication and exosomes derived from hypoxic cardiomyocytes could be effectively engulfed by other cardiomyocytes. However, the precise transfer mechanisms of exosomes are still unclear. In one possible mechanism, exosomes bind to the plasma membrane of the receptor through a specific pathway and release the effectors into the cytoplasm of the receptor cell. In another possible mechanism, exosomes enter receptor cells through endocytosis and fuse with the intima to release effectors [26]. Further exploration of the detailed transfer mechanism of exosomes is required in future studies.

LncRNAs have long been believed to mediate simple transcriptional interference [28], but recent research has confirmed that these molecules are involved in regulating transcription, post-transcriptional processes, epigenetic modifications, histone modification, and protein function [29]. Numerous studies have confirmed that lncRNAs participate in AMI and are regarded as a novel group of regulators of this condition [8–10]. Exosomal lncRNAs have been shown to be involved in the incidence of diseases such as cancer [30], rheumatoid arthritis [31], cholestatic liver injury [32], and atherosclerosis [33]. However, the function and regulation of exosomal lncRNAs in AMI have not been reported. Thus, we isolated exosomes from the serum of AMI patients and healthy controls, identified 65 differentially expressed lncRNAs between the AMI and normal groups using RNA-Seq and identified HCG15 as a new biomarker. HCG15 is located at the 6p21 locus in humans, and there is little related literature on HCG15. Along with these findings, our study provides novel insight into the function of HCG15 and reveals a potential molecule for the early diagnosis of AMI.

Based on clinical observations and animal experiments, apoptosis and inflammation play a vital role in the process of AMI and heart failure [34]. Previous studies have shown that the mitogen-activated protein kinase (MAPK) signaling pathway is stimulated in pathological processes, such as oxidative stress, IR, and inflammation, and plays a crucial role in postinfarct remodeling and heart failure after AMI [35]. c-Jun NH-terminal kinase (JNK), cellular signal-regulated kinase-1/2 (ERK1/2), and p38 MAPK are subfamilies of MAPK signaling pathways that are associated with a range of myocardial pathologies, such as inflammation, apoptosis, cardiac hypertrophy, and heart failure [36]. Activation of the JNK and p38 pathways induces cardiomyocyte apoptosis, dysfunction, and fibrosis [35], while ERK1/2 has a bidirectional role in apoptosis [2]. In this research, we detected molecules of the JNK, ERK1/2 and p38 pathways by western blotting. The results showed that only the level of phosphorylated p38 was increased when AC16 cells were incubated with Exo-H, while other molecules had no significant changes. These results suggested that the p38 MAPK pathway was activated in cardiomyocytes treated with Exo-H. Moreover, the activation was weakened when HCG15 was silenced in AC16 cells. NF-κB is a nuclear transcription factor that regulates gene expression related to inflammation and apoptosis during various pathologies, including AMI [36]. Under normal conditions, NF-κB is mainly located in the cytoplasm in the inactivated state, as it is sequestered by IκB family proteins, including IκB-α. Once stimulated, NF-κB is phosphorylated, and IκB-α is degraded by IKK. NF-κB subunits transfer from the cytoplasm to the nucleus and regulate the expression of inflammatory cytokines [37]. The NF-κB subunit p65 has been shown to be involved in MI [38–40]. We found that the NF-κB/p65 pathway was also activated in cardiomyocytes treated with Exo-H, and reducing HCG15 levels had an antagonistic effect. Moreover, blocking the NF-κB and p38 pathways with specific inhibitors attenuated cell apoptosis and inflammatory cytokine production. In addition, overexpression of lncRNA HCG15 aggravated cardiac IR injury, while blocking the NF-κB and p38 pathways alleviated cardiac IR injury. These results confirmed that lncRNA HCG15 in exosomes released from hypoxic cells mediated myocardial injury through the NF-κB/p65 and p38 pathways, although the detailed process should be further elucidated.

## Conclusions

In summary, lncRNA HCG15 levels were significantly higher in exosomes isolated from AMI patients and hypoxic cells than the controls. HCG15 released by hypoxic cells contributes to cardiomyocyte apoptosis and the production of inflammatory cytokines by activating the NF-κB/p65 and p38 pathways. This study not only helps elucidate the function of exosomal lncRNAs in the pathogenesis of AMI but also contributes to the development of novel therapeutic strategies.

## Supplementary information

sub_Figure 1

Supplementary Tables
