# Supplementary material for: Loss of exosomal LncRNA HCG15 prevents acute myocardial ischemic injury through the NF-κB/p65 and p38 pathways
Source: Cell Death Dis. 2021 Oct 27;12(11):1007. doi: 10.1038/s41419-021-04281-8 (PMC8551195; doi:10.1038/s41419-021-04281-8)
Supplement: Supplementary file 1 — sub_Figure 1 [file 41419_2021_4281_MOESM1_ESM.docx]

**Supplementary Figure**

**
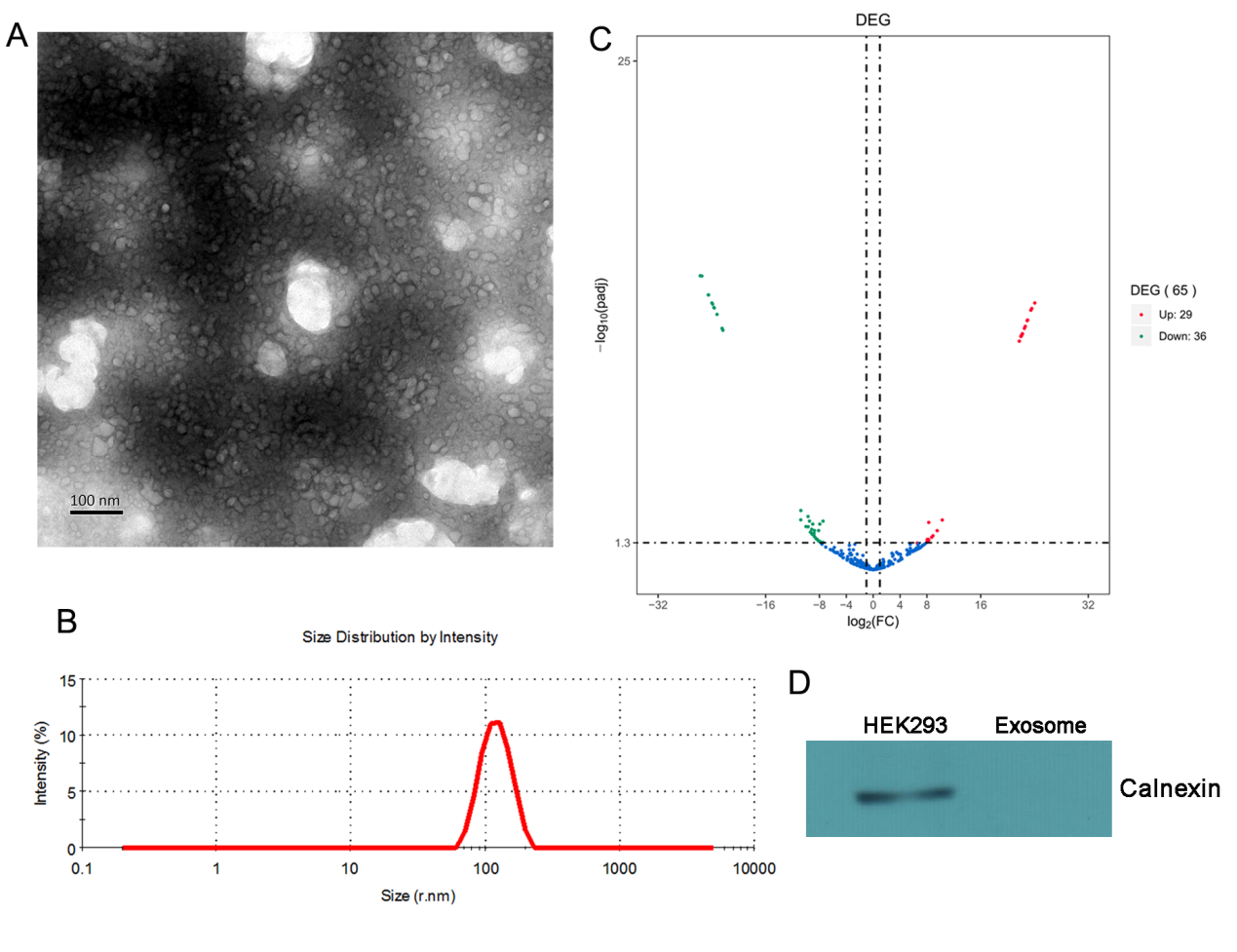
**

**Figure S1. Characterization of exosomal particles and volcano plot of differential lncRNA expression from exosomes**. TEM (A) and particle size (B) analysis of exosomes isolated from the serum of MI patients. (C) Volcano plot of differential exosomal lncRNA expression between MI patients and normal healthy controls. (D) The negative indicator calnexin was detected in the identification of exosomes by Western blots.
