# Supplementary material for: Loss of exosomal LncRNA HCG15 prevents acute myocardial ischemic injury through the NF-κB/p65 and p38 pathways
Source: Cell Death Dis. 2021 Oct 27;12(11):1007. doi: 10.1038/s41419-021-04281-8 (PMC8551195; doi:10.1038/s41419-021-04281-8)
Supplement: Supplementary file 2 — Supplementary Tables [file 41419_2021_4281_MOESM2_ESM.docx]

**Supplementary Tables**

**Supplementary Table 1. Clinical characteristics of enrolled subjects in RNA-Seq.**

**Table 1. Clinical characteristics of enrolled subjects in RNA-Seq.**

| Variable | MI group  （n=6） | Control group （n=6） | *P* Value |
| --- | --- | --- | --- |
| Age (years) | 65.37±12.91 | 60.45±11.72 | 0.505 |
| Male/Female | 3/3 | 3/3 | 1.000 |
| Hypertension (%) | 3 (50.0) | 2 (33.33) | 0.558 |
| DM (%) | 1 (16.67) | 0 (0) | 0.296 |
| Smoking (%) | 2 (33.33) | 2 (33.3) | 1.000 |
| Creatinine (μmol/l) | 115.79±41.7 | 82.19±19.85 | 0.105 |
| HDL (mmol/l) | 1.04±0.27 | 1.15±0.29 | 0.512 |
| LDL-C (mmol/l) | 3.42±1.25 | 2.57±0.92 | 0.209 |
| cTnT (μg/ml) | 643.92±739.39 | 8.24±6.56 | <0.001 |

Results were presented as mean ± standard deviation or n (%). DM, diabetes mellitus; HDL, high-density lipoprotein; LDL, low-density lipoprotein; cTnT, cardiac troponin T.

**Supplementary Table 2: Differentially expressed lncRNAs between the MI and control groups**

**Supplementary Table 2.** Differentially expressed lncRNAs between the MI and control groups

| tracking_id | log2FoldChange | pvalue | padj |
| --- | --- | --- | --- |
| MSTRG.83073 | -25.7474 | 1.01E-17 | 3.53E-15 |
| MSTRG.54677 | -25.4983 | 2.07E-17 | 3.62E-15 |
| MSTRG.74874 | -24.5422 | 3.05E-16 | 3.08E-14 |
| ENSG00000226542 | -24.4916 | 3.52E-16 | 3.08E-14 |
| ENSG00000231031 | -24.0082 | 1.32E-15 | 7.69E-14 |
| MSTRG.13673 | 24.03315 | 1.23E-15 | 7.69E-14 |
| MSTRG.81312 | -23.9052 | 1.74E-15 | 8.71E-14 |
| MSTRG.26301 | -23.7128 | 2.91E-15 | 1.27E-13 |
| MSTRG.74990 | -23.6513 | 3.45E-15 | 1.34E-13 |
| MSTRG.111698 | 23.58735 | 4.09E-15 | 1.43E-13 |
| MSTRG.9051 | 23.44718 | 5.91E-15 | 1.73E-13 |
| MSTRG.72857 | 23.44759 | 5.92E-15 | 1.73E-13 |
| MSTRG.3784 | -23.2368 | 1.03E-14 | 2.78E-13 |
| MSTRG.30826 | 22.96046 | 2.13E-14 | 5.32E-13 |
| MSTRG.60638 | 22.88754 | 2.57E-14 | 5.63E-13 |
| MSTRG.33363 | 22.90607 | 2.45E-14 | 5.63E-13 |
| MSTRG.20712 | 22.61107 | 5.25E-14 | 1.08E-12 |
| MSTRG.48507 | 22.47789 | 7.39E-14 | 1.36E-12 |
| MSTRG.56316 | -22.4966 | 7.05E-14 | 1.36E-12 |
| MSTRG.53250 | -22.3724 | 9.68E-14 | 1.69E-12 |
| MSTRG.75527 | 22.19664 | 1.51E-13 | 2.52E-12 |
| MSTRG.81960 | 22.17174 | 1.61E-13 | 2.55E-12 |
| MSTRG.29349 | 22.15581 | 1.67E-13 | 2.55E-12 |
| MSTRG.65411 | 22.07313 | 2.06E-13 | 3.01E-12 |
| MSTRG.88431 | 22.01386 | 2.34E-13 | 3.15E-12 |
| MSTRG.42476 | 22.03306 | 2.28E-13 | 3.15E-12 |
| ENSG00000268906 | 21.95827 | 2.70E-13 | 3.37E-12 |
| MSTRG.12873 | 21.97913 | 2.61E-13 | 3.37E-12 |
| MSTRG.100559 | 21.72516 | 4.81E-13 | 5.81E-12 |
| MSTRG.87694 | -10.7707 | 0.000107 | 0.001247 |
| MSTRG.49923 | -9.69925 | 0.000217 | 0.002453 |
| MSTRG.32726 | -10.7849 | 0.000329 | 0.003603 |
| MSTRG.62282 | 10.26366 | 0.000345 | 0.003658 |
| MSTRG.93154 | -9.48568 | 0.000412 | 0.004164 |
| MSTRG.5765 | -7.47804 | 0.000416 | 0.004164 |
| MSTRG.51758 | 8.250949 | 0.000497 | 0.004836 |
| MSTRG.73410 | -7.96106 | 0.000616 | 0.005827 |
| MSTRG.52260 | -9.00112 | 0.000634 | 0.005843 |
| ENSG00000283103 | -10.0074 | 0.000862 | 0.007739 |
| MSTRG.109309 | -9.68919 | 0.000898 | 0.007854 |
| MSTRG.77211 | -9.15295 | 0.001228 | 0.010486 |
| MSTRG.109993 | -8.74212 | 0.001485 | 0.01209 |
| MSTRG.75489 | -8.13377 | 0.001481 | 0.01209 |
| MSTRG.96022 | 9.514017 | 0.001537 | 0.012225 |
| MSTRG.87837 | -9.34612 | 0.001861 | 0.014475 |
| MSTRG.26646 | -9.18662 | 0.002125 | 0.016172 |
| MSTRG.26274 | -8.81353 | 0.002221 | 0.016542 |
| ENSG00000253669 | -9.03859 | 0.002597 | 0.018939 |
| MSTRG.208 | -8.86116 | 0.002758 | 0.0197 |
| MSTRG.91531 | 8.882493 | 0.00311 | 0.021769 |
| MSTRG.13816 | -8.72958 | 0.003539 | 0.024284 |
| MSTRG.52658 | 8.682352 | 0.003854 | 0.025938 |
| MSTRG.9945 | -8.4691 | 0.004702 | 0.030479 |
| MSTRG.2058 | -8.48615 | 0.004622 | 0.030479 |
| MSTRG.88424 | 8.14534 | 0.005092 | 0.032404 |
| MSTRG.46816 | 8.169949 | 0.005399 | 0.03321 |
| ENSG00000261094 | -8.34906 | 0.00545 | 0.03321 |
| MSTRG.104259 | 8.032903 | 0.005503 | 0.03321 |
| MSTRG.80979 | 8.250835 | 0.006035 | 0.035799 |
| MSTRG.66155 | -8.18524 | 0.006432 | 0.037522 |
| MSTRG.5624 | -8.04723 | 0.007375 | 0.041634 |
| MSTRG.80968 | -7.91415 | 0.00736 | 0.041634 |
| MSTRG.109498 | 7.952861 | 0.008141 | 0.045228 |
| MSTRG.99089 | -7.48459 | 0.008384 | 0.04585 |
| MSTRG.94600 | 6.543246 | 0.009201 | 0.049546 |

**Supplementary Table 3: Clinical characteristics of enrolled subjects in qRT-PCR**

**Supplementary Table 3. Clinical characteristics of enrolled subjects in qRT-PCR.**

| Variable | MI group  （n=45） | Control group（n=45） | P Value |
| --- | --- | --- | --- |
| Age (years) | 64.93±13.77 | 60.45±11.72 | 0.100 |
| Male/Female | 39/4 | 31/14 | 0.042 |
| Hypertension (%) | 26 (57.8) | 11 (24.44) | 0.001 |
| DM (%) | 7 (15.6) | 5 (11.11) | 0.534 |
| Smoking (%) | 22 ((48.9) | 15 (33.3) | 0.133 |
| Creatinine (μmol/l) | 108.21±42.68 | 82.19±19.85 | 0.0004 |
| HDL (mmol/l) | 1.04±0.26 | 1.15±0.29 | 0.061 |
| LDL-C (mmol/l) | 3.26±1.20 | 2.57±0.92 | 0.003 |
| cTnT (ng/ml) | 1627.43±2780.15 | 6.32±5.61 | <0.001 |

Results were presented as mean ± standard deviation or n (%). DM, diabetes mellitus; HDL, high-density lipoprotein; LDL, low-density lipoprotein; cTnT, cardiac troponin T.
